# The COVID-19 Pandemic and Acute Coronary Syndrome Admissions and Deaths in Allegheny County, Pennsylvania

**DOI:** 10.3390/healthcare13243303

**Published:** 2025-12-16

**Authors:** Brandon M. Herbert, Indu G. Poornima, Suresh R. Mulukutla, Zhen-qiang Ma, LuAnn Brink, Yuefang Chang, Akira Sekikawa, Lewis H. Kuller

**Affiliations:** 1 Department of Epidemiology, School of Public Health, University of Pittsburgh, Pittsburgh, PA 15260, USA; brandon.herbert@pitt.edu (B.M.H.);; 2 Department of Cardiology, Allegheny Health Network-Allegheny General Hospital, Pittsburgh, PA 15212, USA; indu.poornima@ahn.org; 3 Heart and Vascular Institute, UPMC, Pittsburgh, PA 15213, USA; mulukutlasr@upmc.edu; 4 Bureau of Epidemiology, Pennsylvania Department of Health, Harrisburg, PA 17120, USA; zma@pa.gov; 5 Allegheny County Health Department, Pittsburgh, PA 15219, USA; lu.phd.pitt@gmail.com; 6 Department of Neurosurgery, University of Pittsburgh, Pittsburgh, PA 15219, USA; yuc2@pitt.edu

**Keywords:** COVID-19, acute coronary syndrome’ ischemic heart disease death, acute myocardial infarction, epidemiology

## Abstract

**Highlights:**

**What are the main findings?**

- We evaluated the impact of COVID-19 on heart disease hospitalizations and deaths using data from Allegheny County (population 1.2 million) from January 2017 to November 2020.
- During the pandemic, heart disease hospitalizations decreased by 14.8% and heart disease deaths increased by 2.4%

**What are the implications of the main findings?**

- The COVID-19 pandemic minimally impacted overall heart disease trends.
- Interpreting changes in heart disease hospitalizations and death during the pandemic must be done in the context of long-term trends.

**Abstract:**

**Background/Objectives**: This study evaluated the impact of the COVID-19 pandemic on trends of acute coronary syndrome hospitalizations, all-cause deaths, and ischemic heart disease (IHD) deaths in Allegheny County, Pennsylvania. **Methods**: Inpatient hospital records from two hospital systems within Allegheny County, Pennsylvania, were aggregated from January 2017 to November 2020. The primary diagnoses were acute myocardial infarction (AMI) and unstable angina. The Pennsylvania Department of Health provided all-cause and IHD death counts for the same period. We compared absolute percentage changes in admissions by year (March–November) and trends by age-specific groups (<45, 45–64, 65–74, ≥75) from the pre-pandemic (January 2017–February 2020) to pandemic (March 2020–November 2020) period using an interrupted time-series analysis. **Results**: There were 11,913 AMI hospitalizations pre-pandemic and 2170 AMI hospitalizations during the pandemic period. AMI hospitalizations decreased by 14.8% and unstable angina hospitalizations decreased by 30.7% during the pandemic compared to 2019, with the largest decreases occurring in those aged ≥75. Total mortality increased by 9.2%, and IHD mortality increased by 2.4%. About 80% of the increase in deaths was due to COVID-19, and approximately 75% of deaths occurred in those aged ≥75 and in long-term care facility residents. **Conclusions**: The COVID-19 pandemic did not markedly alter the longitudinal declining trend of AMI hospitalizations and IHD deaths in Allegheny County.

## 1. Introduction

The effects of the severe acute respiratory syndrome coronavirus 2 (SARS-CoV-2), the virus responsible for the coronavirus disease 2019 (COVID-19) pandemic, on acute myocardial infarction (AMI) hospital admissions, treatment, and death due to cardiovascular disease (CVD) have been reported by studies in the United States (US) and other countries [1,2,3]. On 11 March 2020, the World Health Organization declared COVID-19 a global pandemic. By the end of 2020, over 80 million confirmed cases and 1.8 million deaths had been reported worldwide [4]. While the systemic effects of the pandemic have been documented in conditions ranging from viral hepatitis [5] to long-term immune responses [6], its impact on acute cardiovascular presentations remains a critical area of study.

Most studies have reported a decline in hospitalizations for AMI, especially at the beginning of the pandemic during the lockdowns [7,8,9,10,11,12,13,14,15,16,17,18,19,20,21,22,23]. A recent report noted a significant decline in overall cardiovascular hospitalizations for the fee-for-service Medicare population. Much of the decline occurred during the lockdown phases of the pandemic, with a gradual return to pre-pandemic levels of hospitalizations [22]. Studies have also reported reduced diagnostic and therapeutic interventions [15,24,25,26,27,28,29], as well as increases in cardiovascular and total mortality, especially among older individuals and those in long-term care facilities (LTCFs) [30,31,32,33,34,35]. Most of these were single-center studies and may not apply to a defined community. Additionally, many have not considered longitudinal trends in AMI admissions prior to the COVID-19 pandemic but, rather, have compared 2019 with 2020 or examined only monthly changes during the pandemic.

Hospitalizations for AMI in the US and other countries have been declining fairly dramatically over the past 10–15 years [36,37,38,39,40]. For context, national data indicate that AMI hospitalization rates have decreased by over 20% in recent decades [36]. A decline in AMI in 2020 as compared to 2019 would not be unexpected as part of the trend in declining admissions for AMI. Hospitals and communities that had a very high burden of COVID-19 reducing the amount of available hospital beds, especially intensive care unit (ICU) beds and staff, may report a greater decline in AMI admissions and diagnostic procedures as compared to hospitals in other communities that had a greater availability of healthcare services to deal with the pandemic. It is possible that in several communities, such as in specific counties or states, total hospitalizations for AMI may not have changed very much, only the distribution of cases among the hospitals in relation to the availability of hospital beds and staff. Also, policies regarding admissions of individuals in LTCFs to hospitals during the pandemic for treatments of AMI may vary.

We aimed to bridge this gap by integrating long-term pre-pandemic trends with community-wide data. We have identified practically all admissions for AMI and unstable angina in the two primary healthcare systems in Allegheny County, Pennsylvania (PA), which provide most of the hospital care for the 1.2 million people in the county. The availability of similar data from two distinct healthcare systems with different data collection procedures provides a validation of the observations in the study. This study was also able to analyze county all-cause death trends, ischemic heart disease death trends, and COVID-19 cases and deaths among Allegheny County independent residents and LTCF residents.

The specific aims of this study were: (1) To evaluate trends in acute coronary syndrome (ACS) admissions before and during the pandemic across sex, age group, and primary diagnosis; and (2) To evaluate age-specific all-cause and ischemic heart disease mortality trends during the same period.

## 2. Materials and Methods

### 2.1. Study Design and Population

Medical records were accessed from the only two hospital systems in Allegheny County, PA, which provide most of the medical care in the county, eight hospitals in one system and five in the other. Admissions for acute coronary syndrome were obtained from 1 January 2017 to 30 November 2020. Patients with a primary discharge diagnosis of non-ST segment elevation myocardial infarction (NSTEMI), ST segment elevation myocardial infarction (STEMI), or unstable angina were included in the study population and were identified using ICD-10 codes. ICD-10 codes for these diagnoses are included in Table S1.

Patients were excluded from analyses if they died in general inpatient hospice status, were transferred into a facility where mortality quickly occurred and little medical care was provided, or their age was <18 years. These exclusions were applied to prevent double-counting of transfers and to focus on acute presentations managed within the hospital system. All data was deidentified and admission dates were truncated to month and year. The University of Pittsburgh Institutional Review Board (IRB # STUDY20080150) and Allegheny Health Network (IRB #2020-230) reviewed this study and determined that it meets the regulatory requirements for exempt research under 45 CFR 46.104, Secondary research on data (no consent required), and followed the Strengthening the Reporting of Observational Studies in Epidemiology (STROBE) guidelines.

Patient information including age, sex, race, discharge status, and in-hospital death was collected from both health systems. Admissions were aggregated into annual (March–November) and monthly counts and patients were stratified into four pre-defined age groups: <45, 45–64, 65–74, and ≥75. Patients with missing data for age or sex were included in total counts but excluded in sex- and age-specific analyses.

For population counts of Allegheny County, the US Census Bureau QuickFacts website and American Community Survey Demographic and Housing Estimates were accessed [38,39]. Recent US Census reports showed only a 2% change in the population of Allegheny County, PA, from 2010 to 2020, indicating a stable population structure for the duration of the study. Data was obtained from the PA Department of Health regarding age-specific all-cause deaths where ischemic heart disease (ICD-10 code: I20*-I25*) was the underlying cause of death between 2017 and 2020. Mortality data was available through December 2020, whereas hospital admission data was limited to November 2020 due to reporting lags. Age-specific COVID-19 cases and deaths among both independent residents and LTCF residents were obtained from the Allegheny County Health Department.

### 2.2. Statistical Analyses

Total and sex-specific admissions were summed for the pre-pandemic, January 2017 to February 2020, and pandemic, March 2020 to November 2020, periods. Demographics for AMI admissions are described in Table 1. Continuous variables are expressed as means with standard deviations and categorical variables as frequencies and proportions. Annual (March–November) admissions for AMI and unstable angina were plotted and independent *t*-tests with pooled variances were used to compare mean monthly admissions to the preceding year (Table S2). March-to-November admissions were analyzed to account for seasonality in comparisons of the COVID-19 period to the pre-pandemic period.

Trends in monthly admission counts were compared pre-pandemic to pandemic using an interrupted time-series analysis [41,42]. Coefficients were estimated by ordinary least squares regression and Newey–West standard errors were produced to account for autocorrelation (lag of 1) and possible heteroscedasticity [41,42]. Interrupted time-series analyses were performed across sex and age groups to obtain pre-pandemic slopes, changes in starting level of the pandemic slope relative to the pre-pandemic slope, and changes in pandemic slopes relative to pre-pandemic slopes. All models accounted for autocorrelation with a lag of 1. Similar models were constructed for all-cause and ischemic heart disease deaths. The interrupted time-series analysis was conducted with Stata 16.1.41. Figures and simple linear regression slopes were created with GraphPad Prism version 9.1.2 for Windows, GraphPad Software, San Diego, CA, USA, www.graphpad.com.

## 3. Results

The population estimate for Allegheny County in 2019 was 1,221,744, with 18.5% of the population 65 years or older, 52.3% women, and 79.9% non-Hispanic Whites. The median household income was USD 61,043, with 10.8% of the population in poverty [43,44]. Other demographics for the Allegheny County population are provided in Table S3.

During the pandemic period, March to December 2020, there were 53,906 COVID-19 cases reported to the ACHD, and 4418 of these cases resided in LTCFs (Table S4). Among all residents, the majority of COVID-19 cases were among those aged <45 (29,126 cases, 54.0%) with cases peaking in July and December. There were 1414 COVID-19 deaths among all residents from March to December, with the greatest proportion occurring in those aged ≥75 (1071 deaths, 75.7%) (Table S4). The majority of COVID-19 deaths occurred in LCTFs (893 deaths, 63.2%).

There were 11,913 admissions for AMI in the pre-pandemic period and 2170 admissions during the pandemic period (Table 1). The number of hospitalizations for AMI and cases of COVID-19 during the pandemic were similar. Admissions for AMI during the pandemic tended to be younger, with a greater proportion of admissions occurring in the 45–64 age group (33.7% pre-pandemic vs. 36.4% pandemic) and similar reported rates of in-hospital mortality (5.8% pre-pandemic vs. 6.7% pandemic). There were some missing data for age, sex, and race, largely during the pre-pandemic period. The proportion of patients missing age or sex data was low (3.1% and 3.6% of the pre-pandemic population, respectively).

Total annual March–November AMI or unstable angina admissions and annual percentage changes from year to year within Allegheny County, PA, are shown in Table S2. Annual AMI admissions declined 10.3%, 9.3%, and 14.8% between 2017 and 2018, 2018 and 2019, and 2019 and 2020 during the pandemic. On the other hand, unstable angina admissions decreased by 9.6%, 4.0%, and 30.7% over the same time periods. Mean monthly admissions for AMI were significantly lower in 2020 compared to 2019, as were unstable angina admissions.

**Figure 1:**
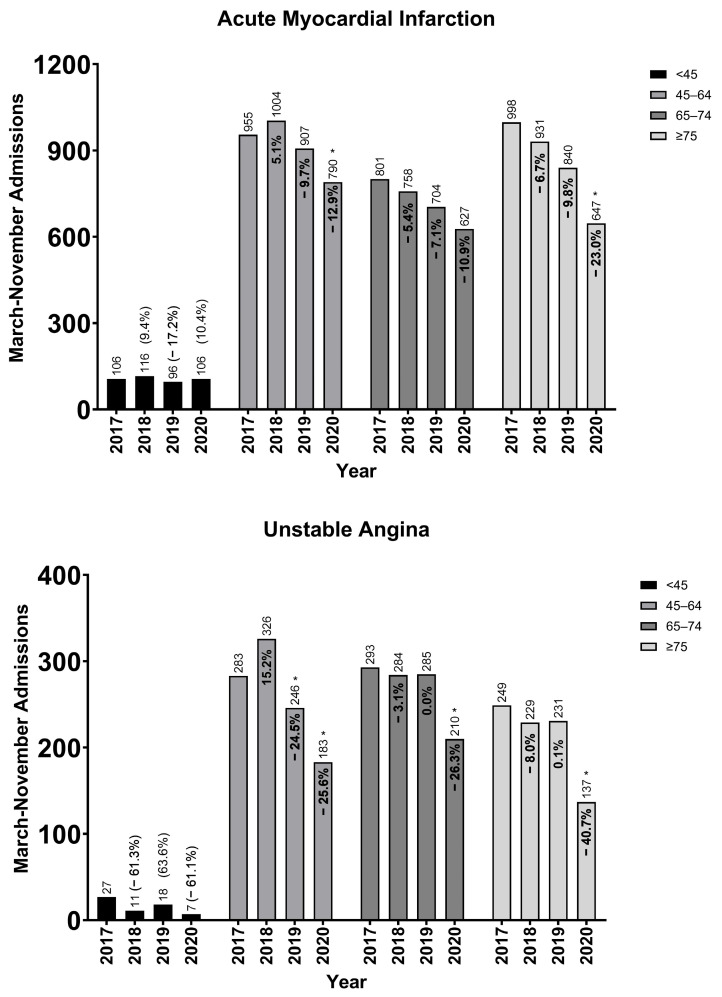
Age-specific annual (March–November) acute myocardial infarction and unstable angina admissions at the two hospital systems in Allegheny County, Pennsylvania. Age-specific annual (March–November) admissions for acute myocardial infarction and unstable angina with annual percentage change. Admissions aggregated across both health systems. * Significant (*p* < 0.05) difference in mean monthly admissions compared to preceding year (independent *t*-test with pooled variances).

Annual AMI admissions decreased by 12.9% in 2019–20 in the 45–64 age group, 10.9% in the 65–74 age group, and 23.0% in the ≥75 age group (Figure 1).

Declines were similar among men and women for specific age groups (Figure S1). The greatest drop in AMI admissions occurred at the beginning of the pandemic during the lockdown between March and April 2020 (22.6%), consistent with previous reports in the literature (Figure S2). However, the number of admissions per month during 2020 rebounded between April and May 2020 (Figure S2).

All-cause mortality increased by 9.2% between 2019 and 2020; by 5.4% in the 45–64 age group, 16.4% in the 65–74 age group, and 8.0% in the ≥75 age group (Figure S3). Only the overall increase in all-cause mortality, and among the 65–74 age group, was statistically significant from 2019 to 2020. Ischemic heart disease mortality, on the other hand, increased non-significantly by 2.8%; 11.1% in the 45–64 age group, 19.5% in the 65–74 age group, and decreased by 5.1% in the ≥75 age group between 2019 and 2020. None of these changes in ischemic heart disease as the underlying cause of death were statistically significant (Figure S3).

**Figure 2:**
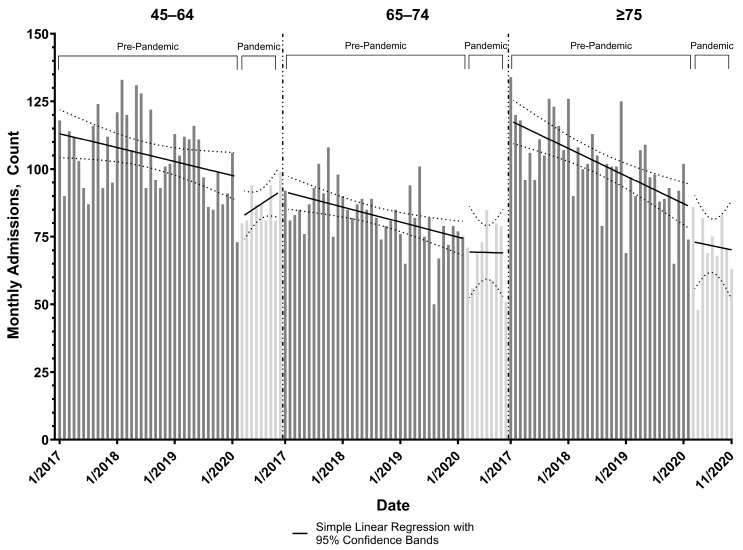
Age-specific monthly acute myocardial infarction admissions at the two hospital systems in Allegheny County, Pennsylvania.

Pre-pandemic slopes of AMI admissions in the 45–64, 65–74, and ≥75 age groups were statistically non-zero with negative trends (−0.43, −0.46, −0.85 admissions/month, respectively) (Table 2, Figure 2). Only among the 45–64 age group was a significant decrease in AMI admissions detected at the onset of the pandemic (−14.35 admissions, 95% confidence interval (CI): −24.48, −4.22).

Across all age groups, the trend in all-cause deaths increased during the pandemic but was only significant among the 65–74 age group (10.8 deaths/month, 95% CI: 1.93, 19.59) (Table S5). All pre-pandemic slopes for monthly ischemic heart disease deaths did not significantly deviate from zero (Table S6). Among the aggregated data that included all age groups, the post-pandemic slope of deaths differed significantly from the pre-pandemic slope where an increase in 5.38 deaths/month was detected (95% CI: 0.66, 10.10). A significant increase in the trend for monthly ischemic heart disease deaths was found in the 65–74 age group (2.15 more deaths/month, 95% CI: 1.28, 3.02).

The black population in Allegheny County, PA, is relatively small, as noted in Table S3. Mean monthly AMI admissions for the black population declined by approximately 22.6% (95% CI: −24.2%, −21.6%) in 2020 as compared to 2019, while the white population experienced a decline of 13.8% (95% CI: −16.2%, −10.6%) over the same period (Figure S4).

## 4. Discussion

This study evaluated hospitalizations for AMI and unstable angina, CVD mortality, and COVID-19 cases and deaths for a defined community that includes hospitals that provided most of the hospital care. Previous studies of the trends in AMI and CVD in the US have included specific health insurance populations, such as the Northern California Kaiser Permanente, which initially reported a large decrease in hospitalizations for AMI early in the pandemic, but subsequent analysis showed no or little change in hospitalizations for AMI [37,45,46].

The findings from this study extend on previous analyses of the impact that the COVID-19 pandemic has had on CVD admissions. Studies performed in the US have described drops in admissions for acute coronary syndrome of 40% and marked declines in acute cardiovascular hospitalization of >40% during the first months of the pandemic [7,8,9,11]. A systematic review of the impact of the COVID-19 pandemic on the care and management of patients with acute CVD concluded that substantial decreases in total admissions, procedures, and length of stay were consistent across 27 studies investigating acute coronary syndromes, stroke, and other types of CVD [1].

A recent report evaluated Medicare hospitalizations during the pandemic [22]. Hospitalizations for AMI or stroke were reduced by 14% from levels in 2018–2019. There was a very large decrease in admissions during the initial closure period due to the pandemic in March–April 2020. There were no significant gender differences in the reduction in admissions. A study from New York City using the four-hospital NYU system noted a substantial decrease in many different causes of hospitalizations during the peak of the COVID-19 pandemic, including a decrease in circulatory system admissions by almost 40% with a gradual return in admissions to prior pandemic levels [21]. In western Massachusetts, the Baystate Health System found there was no decline in MI admissions [47]. Similarly, a study in Taiwan found that there was no evidence of a decrease in STEMI admissions [48]. A study in St. Joseph’s Health System in the northwestern United States reported an initial substantial decrease in NSTEMI hospitalizations during the COVID-19 pandemic, a small decrease in STEMI, and a gradual return to pre-COVID-19 pandemic levels over time [49].

A study from the Cleveland Clinic suggests a substantial decrease in referrals to the clinic for CVD during the early phase of the COVID-19 pandemic [11]. This may be due to the Cleveland Clinic primarily being a referral center and patients preferring to stay in their communities during the pandemic. Similarly, there was a 43% decrease in the large tertiary care Mass General Brigham Health System in Massachusetts during the acute phase of the pandemic [7]. A study that evaluated medical care for coronary artery disease provided by a large number of hospitals in the US also reported about a 20% decrease in AMI cases [8]. Other studies have reported a decrease in CVD procedures, especially elective procedures, which may be consistent with the decline in unstable angina noted in this study due to a decrease in screening [1,2,50]. Our findings of a 30.7% decline in unstable angina admissions are particularly notable. This substantial drop may reflect a reduction in elective testing and screening procedures during the pandemic, consistent with reports of decreased elective cardiovascular procedures. Additionally, we observed that the black population in Allegheny County experienced a larger relative decline in mean monthly AMI admissions (22.6%) compared to the white population (13.8%) in 2020 versus 2019. This disparity may reflect differential access to care or hesitation to seek medical attention during the pandemic, highlighting a potential area for health equity intervention.

A population-based study performed in Berlin, Germany, reported results very similar to this Allegheny County, PA, study, with only a very small decrease in admissions for AMI [51]. Our results align with international findings. A study from Sweden found that while AMI presentations dropped, the incidence of AMI comorbidity with COVID-19 was significant [52]. These international comparisons suggest that the phenomenon of reduced ACS presentations was global, likely driven by patient behavior and system reconfiguration rather than a biological reduction in disease incidence.

The majority of studies in the US have compared data from 2020 to the same period in 2019. This approach presumes that there has been little change in AMI prior to 2019. However, we have that shown in Allegheny County, PA, a fairly substantial decline in hospital admissions for AMI between 2017 and 2019 was observed, especially for NSTEMI. Additionally, we have analyzed counts, rather than rates, due to little change in population size in Allegheny County over the study years.

The changes in AMI hospitalizations prior to the pandemic could be due to a decrease in readmissions for individuals who have had a prior AMI or decrease in the incidence of first AMI. We do not have information to further evaluate these two possibilities. Studies of the decline in AMI admissions prior to the COVID-19 pandemic have suggested that there had been a decrease both in first events, i.e., incidence, and in readmissions [35,36,37,38,39,40].

The observed long-term decline in AMI hospitalizations prior to the pandemic is likely driven mainly by reduced incidence resulting from improved primary prevention and risk factor control [53]. Improved acute and outpatient care has historically contributed more to lower case fatality and shifting case mix than to the drop in admissions. Furthermore, policy changes such as the Medicaid expansion in Pennsylvania in 2015 may have influenced these pre-pandemic trends by gradually increasing access to outpatient care, thereby impacting emergency department utilization rates over the study period [54].

Additionally, it is possible that AMIs were being misclassified as COVID-19 hospitalizations. Future research could compare admission diagnoses for COVID-19, AMI, and other CVD with subsequent discharge diagnoses and then evaluate clinical records to determine how many of the COVID-19 hospitalizations have evidence consistent with AMIs that occurred prior to or during the hospitalizations. COVID-19 has adverse effects on the cardiovascular system, but evidence is still being gathered regarding the influence of COVID-19 infection on risk for subsequent AMI [55]. A recent cohort study from Sweden identified that those diagnosed with COVID-19 had over three times the odds of developing AMI within two weeks following their COVID-19 diagnosis compared to those without a history of COVID-19 [56].

This study has several strengths. First, we accounted for longitudinal trends in hospitalizations and deaths by using an interrupted time-series analysis which is a robust and quasi-experimental research design. This approach was important for assessing AMI trends in particular as we observed annual declines of about 10% year-over-year prior to 2020. Second, we analyzed ACS hospitalizations, all-cause deaths, ischemic heart disease deaths, and COVID-19 cases and deaths in a defined population over similar time periods. Lastly, we collected admission data from two separate health systems, which provides strength to the validity of our findings.

Study Limitations—Importantly, several limitations exist in this study. First, the population of Allegheny County is not representative of the entire US and the burden of the COVID-19 pandemic may have been different when compared to other counties. COVID-19 response measures implemented by the state of Pennsylvania, Allegheny County, and local health systems may have contributed to the findings, which may not be similar to responses by other states and localities. Second, distinguishing between a primary AMI and myocardial injury secondary to COVID-19 infection is difficult using ICD-10 codes alone; future chart reviews could refine these counts. Third, our limited sample size may not have detected significant changes in trends of ACS hospitalizations across sex- and age-specific groups, especially since we aggregated data into monthly admissions. Lastly, our data availability for hospital admissions ended in November 2020, meaning our analysis captures the initial waves but may miss later pandemic trends. Larger, nationally representative analyses are needed to understand the impact of the COVID-19 pandemic on ACS admissions, all-cause deaths, and ischemic heart disease deaths across the US.

## 5. Conclusions

During the COVID-19 pandemic in Allegheny County, PA, AMI admissions declined and all-cause mortality increased, but time-trend analyses showed that the pandemic did not markedly alter the pre-existing longitudinal declining trend in AMI admission. The number of hospitalizations for AMI and COVID-19 were similar in magnitude during the pandemic. The largest decline was for unstable angina admissions. The decline in AMI admissions is likely a function of size of COVID-19 pandemic in the community, availability of hospital beds and resources, policies regarding hospitalizations of residents from nursing homes and LTCFs, and availability of medical care within the community to treat CVD. These findings underscore the importance of maintaining continuity of cardiac care and public confidence in hospital safety during public health emergencies to prevent collateral cardiovascular harm.

## Figures and Tables

**Table 1:** Total admissions for acute myocardial infarction during the pre-pandemic ^1^ and pandemic periods at the two hospital systems in Allegheny County, Pennsylvania.

	Total		Men		Women	
	Pre-Pandemic n = 11,913	Pandemic n = 2170	Pre-Pandemic n = 7006	Pandemic n = 1351	Pre-Pandemic n = 4482	Pandemic n = 819
Age, years (mean, SD)	68.5 (13.4)	67.4 (13.3)	66.8 (13.1)	65.8 (12.9)	71.0 (13.5)	70.0 (13.5)
Age Group, years (n, %)						
<45	442 (3.7)	106 (4.9)	306 (4.4)	70 (5.2)	134 (3.0)	36 (4.4)
45–64	4016 (33.7)	790 (36.4)	2748 (39.2)	555 (41.1)	1257 (28.1)	235 (28.7)
65–74	3174 (26.6)	627 (28.9)	1944 (27.8)	390 (28.9)	1215 (27.1)	237 (28.9)
≥75	3909 (32.8)	647 (29.8)	2008 (28.7)	336 (24.9)	1876 (41.9)	311 (38.0)
Missing	372 (3.1)	0 (0)	0 (0)	0 (0)	0 (0)	0 (0)
Sex (n, %)						
Men	7006 (58.8)	1351 (62.3)	-	-	-	-
Women	4482 (37.6)	819 (37.7)	-	-	-	-
Missing	425 (3.6)	0 (0)	-	-	-	-
Race (n, %)						
White	9950 (83.5)	1882 (86.7)	6179 (88.2)	1186 (87.8)	3771 (84.1)	696 (85.0)
Non-White	1244 (10.4)	230 (10.6)	622 (8.9)	123 (9.1)	622 (13.9)	107 (13.1)
Missing	719 (6.0)	58 (2.7)	205 (2.9)	42 (3.1)	89 (2.0)	16 (2.0)

^1^ Pre-pandemic defined as January 2017 to February 2020; Pandemic defined as March 2020 to November 2020.

**Table 2:** Interrupted time-series analysis results modeling monthly acute myocardial infarction admissions by age group at the two hospital systems in Allegheny County, Pennsylvania.

Age Group	Coefficient	Coefficient V95.	95% Confidence Interval
All	Pre-Pandemic ^1^ Slope	−2.92	−3.64, −2.21
	Change in Admission Volume at Start of Pandemic	−20.50	−51.91, 10.91
	Post-Pandemic ^1^ Slope Minus Pre-	4.21	−0.75, 9.17
<45	Pre-Pandemic ^1^ Slope	−0.01	−0.11, 0.10
	Change in Admission Volume at Start of Pandemic	−2.71	−6.35, 0.94
	Post-Pandemic ^1^ Slope Minus Pre-	0.74	0.18, 1.30
45–64	Pre-Pandemic ^1^ Slope	−0.43	−0.85, −0.01
	Change in Admission Volume at Start of Pandemic	−14.35	−24.48, −4.22
	Post-Pandemic ^1^ Slope Minus Pre-	1.60	0.33, 2.86
65–74	Pre-Pandemic ^1^ Slope	−0.46	−0.77, −0.15
	Change in Admission Volume at Start of Pandemic	−4.52	−17.39, 8.35
	Post-Pandemic ^1^ Slope Minus Pre-	0.41	−2.41, 3.24
≥75	Pre-Pandemic ^1^ Slope	−0.85	−1.23, −0.47
	Change in Admission Volume at Start of Pandemic	−12.40	−27.78, 2.98
	Post-Pandemic ^1^ Slope Minus Pre-	0.48	−1.88, 2.85

All models accounted for autocorrelation with a lag of 1; ^1^ Pre-Pandemic defined as January 2017 to February 2020; Pandemic defined as March 2020 to November 2020. Age-specific monthly acute myocardial infarction admissions during the pre-pandemic (January 2017 to February 2020) and post-pandemic (March 2020 to November 2020) periods with overlying simple linear regression and 95% confidence bands performed by age group and time period.

## Data Availability

The data that support the findings of this study are not publicly available due to restrictions related to patient privacy, institutional review board approvals, and data use agreements with participating hospital systems and state health authorities. De-identified data may be made available from the corresponding author upon reasonable request and subject to approval by the data-providing institutions and authors.

## Supplementary Materials

The following supporting information can be downloaded at https://www.mdpi.com/article/10.3390/healthcare13243303/s1. Figure S1: Annual (March–November) age- and sex- specific admissions at the two hospital systems in Allegheny County, Pennsylvania; Figure S2: Monthly acute myocardial infarction admissions in 2020 at the two hospital systems in Allegheny County, Pennsylvania; Figure S3: Age-specific annual (March–November) all-cause and ischemic heart disease deaths in Allegheny County, Pennsylvania; Figure S4: Race-specific acute myocardial infarction admissions at the two hospital systems in Allegheny County, Pennsylvania; Table S1: ICD-10 mapping for research diagnoses; Table S2: Annual (March–November) acute myocardial infarction and unstable angina admissions with annual percentage change at the two hospital systems in Allegheny County, Pennsylvania; Table S3: 2019 US Census Bureau American Community Survey demographics for Allegheny County, Pennsylvania; Table S4: Monthly and total COVID-19 cases and deaths across all residents in Allegheny County, Pennsylvania, by age in 2020; Table S5: Interrupted time-series analysis results modeling monthly all-cause death by age group; Table S6: Interrupted time-series analysis modeling monthly cause of death ischemic heart disease by age group.

## Abbreviations

The following abbreviations are used in this manuscript:

ACHD	Allegheny County Health Department
ACS	Acute Coronary Syndrome
AMI	Acute Myocardial Infarction
CI	Confidence Interval
COVID-19	Coronavirus Disease 2019
CVD	Cardiovascular Disease
ICD-10	International Classification of Diseases, Tenth Revision
ICU	Intensive Care Unit
IRB	Institutional Review Board
LTCF	Long-Term Care Facility
NSTEMI	Non-ST Segment Elevation Myocardial Infarction
PA	Pennsylvania
STEMI	ST Segment Elevation Myocardial Infarction
STROBE	Strengthening the Reporting of Observational Studies in Epidemiology
US	United States
