# Supplementary material for: The COVID-19 Pandemic and Acute Coronary Syndrome Admissions and Deaths in Allegheny County, Pennsylvania
Source: Healthcare (Basel). 2025 Dec 16;13(24):3303. doi: 10.3390/healthcare13243303 (PMC12733221; doi:10.3390/healthcare13243303)
Supplement: Supplementary file 1 [file healthcare-13-03303-s001.zip › Supplementary Figures.pdf]

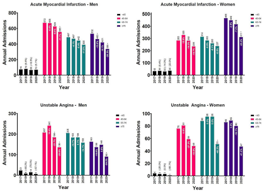

Acute Myocardial Infarction - Men
Acute Myocardial Infarction - Women
Unstable Angina - Men
Unstable Angina - Women
Annual Admissions
Year
2017
2018
2019
2020
<45
45-64
65-74
≥75

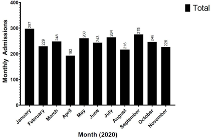

Total
Monthly Admissions
400
300
200
100
0
297
229
248
192
260
243
264
216
275
246
226
January
February
March
April
May
June
July
August
September
October
November
Month (2020)

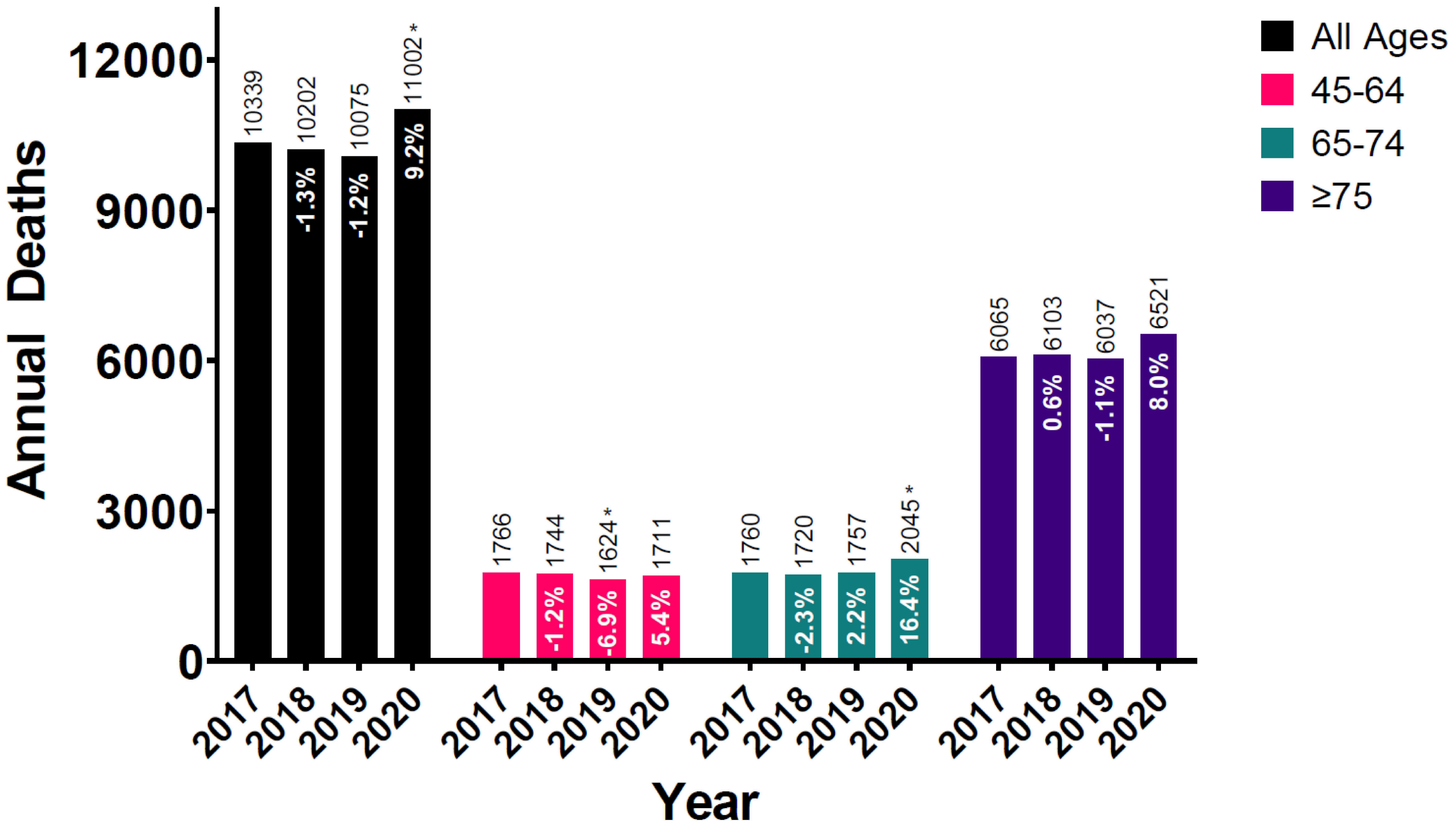
Annual Deaths
12000
9000
6000
3000
0
10339
10202
10075
11002 *
-1.3%
-1.2%
9.2%
1766
1744
1624 *
1711
-1.2%
-6.9%
5.4%
1760
1720
1757
2045 *
-2.3%
2.2%
16.4%
6065
6103
6037
6521
0.6%
-1.1%
8.0%
2017 2018 2019 2020
Year
All Ages
45-64
65-74
≥75

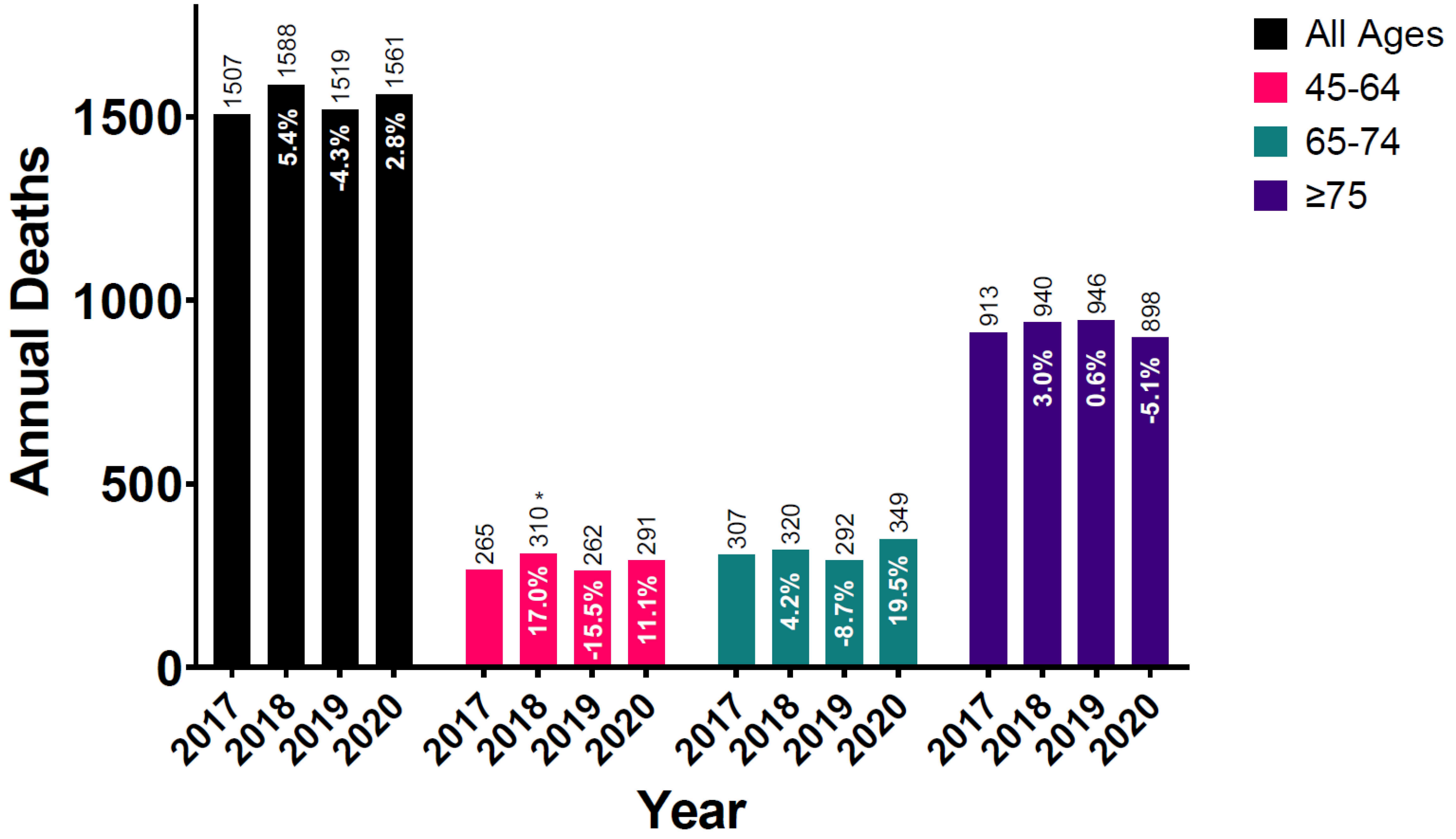
Annual Deaths
1500
1000
500
0
1507
1588
1519
1561
5.4%
-4.3%
2.8%
265
310 *
262
291
17.0%
-15.5%
11.1%
307
320
292
349
4.2%
-8.7%
19.5%
913
940
946
898
3.0%
0.6%
-5.1%
2017 2018 2019 2020
Year
All Ages
45-64
65-74
≥75

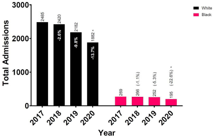

3000
2000
1000
0
Total Admissions
2485
2420
-2.6%
2182
-9.8%
1882 *
-13.7%
269
266
(-1.1%)
252
(-5.3%)
195
(-22.6%) *
2017
2018
2019
2020
2017
2018
2019
2020
Year
White
Black

Supplementary Figure Legends

Figure S1. Annual (March-November) Age- and Sex- Specific Admissions at the Two Hospital Systems in Allegheny County, Pennsylvania.

Sex- and age-specific annual (March-November) acute myocardial infarction and unstable angina admissions with annual percentage change. *Significant ($p<0.05$) difference in mean monthly admissions compared to preceding year (independent t-test with pooled variances).

Figure S2. Monthly Acute Myocardial Infarction Admissions in 2020 at the Two Hospital Systems in Allegheny County, Pennsylvania.

Counts of admissions are aggregated across both health systems.

Figure S3. Age-Specific Annual (March-November) All-Cause and Ischemic Heart Disease Deaths in Allegheny County, Pennsylvania.

Age-specific annual (March-November) all-cause death (top) and ischemic heart disease (I20*-I25*) (bottom) death counts in Allegheny County, Pennsylvania with annual percentage change. *Significant ($p<0.05$) difference in mean monthly deaths compared to preceding year (independent t-test with pooled variances).

Figure S4. Race-Specific Acute Myocardial Infarction Admissions at the Two Hospital Systems in Allegheny County, Pennsylvania.

Race-specific aggregated annual (March-November) acute myocardial infarction admissions with annual percentage change. *Significant ($p<0.05$) difference in mean monthly admissions compared to preceding year (independent t-test with pooled variances).
