# Supplementary material for: The COVID-19 Pandemic and Acute Coronary Syndrome Admissions and Deaths in Allegheny County, Pennsylvania
Source: Healthcare (Basel). 2025 Dec 16;13(24):3303. doi: 10.3390/healthcare13243303 (PMC12733221; doi:10.3390/healthcare13243303)
Supplement: Supplementary file 1 [file healthcare-13-03303-s001.zip › Supplementary Tables.pdf]

Supplementary Tables and Figures

The COVID-19 Pandemic and Acute Coronary Syndrome Admissions and Deaths in Allegheny County, Pennsylvania

Brandon M. Herbert, PhD[1], Indu G. Poornima, MD[2], Suresh R. Mulukutla, MD[3], Zhen-qiang Ma, MD[4], LuAnn Brink, PhD[5], Yuefang Chang, PhD[6], Akira Sekikawa, MD, PhD[1*], Lewis H. Kuller, MD, DrPH[1†]

[1] Department of Epidemiology, School of Public Health, University of Pittsburgh, Pittsburgh, PA; brandon.herbert@pitt.edu (BMH); akira@pitt.edu (AS)
[2] Department of Cardiology, Allegheny Health Network-Allegheny General Hospital, Pittsburgh, PA; indu.poornima@ahn.org
[3] Heart and Vascular Institute, UPMC, Pittsburgh, PA; mulukutlasr@upmc.edu
[4] Bureau of Epidemiology, Pennsylvania Department of Health, Harrisburg, PA; zma@pa.gov
[5] Allegheny County Health Department, Pittsburgh, PA; lu.phd.pitt@gmail.com
[6] Department of Neurosurgery, University of Pittsburgh, Pittsburgh, PA; yuc2@pitt.edu
* Correspondence: akira@pitt.edu
[†] **Deceased**

Table S1: ICD-10 mapping for research diagnoses.

Research Diagnosis	ICD-10 Codes
Non-ST elevation myocardial infarction	I21.4, I21.A1, I22.2
ST elevation myocardial infarction	I21.01, I21.02, I21.09, I21.11, I21.19, I21.21, I21.29, I21.3, I22.0, I22.1, I22.8, I22.9
Unstable angina	I20.0, I25.110, I25.700, I25.710, I25.720, I25.730, I25.750, I25.760, I25.790

Table S2: Annual (March-November) acute myocardial infarction and unstable angina admissions with annual percentage change at the two hospital systems in Allegheny County, Pennsylvania.

**Year**	**Acute Myocardial Infarction** n (% change from prior year)	**Unstable Angina** n (% change from prior year)
2017	3,131	691
2018	2,809 (-10.3%)[1]	625 (-9.6%)
2019	2,547 (-9.3%)	600 (-4.0%)
2020	2,170 (-14.8%)[1]	416 (-30.7%)[1]

[1]Significant (p<0.05) difference in mean monthly admissions compared to preceding year (independent t-test with pooled variances).

Table S3: 2019 US Census Bureau American Community Survey demographics for Allegheny County, Pennsylvania.

	**Estimate**	**Percent**
Total Population	1,221,744	
Men	590,342	48.3%
Women	631,402	51.7%
Sex ratio (men per 100 women)	91.5	
45 to 54 years	151,346	12.4%
55 to 59 years	88,829	7.3%
60 to 64 years	88,320	7.2%
65 to 74 years	123,499	10.1%
75 to 84 years	66,257	5.4%
85 years and over	36,368	3.0%
Median age (years)	40.8	
White	976,172	79.9%
Black or African American	157,060	12.9%
Asian	45,389	3.7%
Two or more races	35,305	2.9%

Table S4: Monthly and Total COVID-19 cases and deaths across all residents in Allegheny County, Pennsylvania by age in 2020.

	**CASES**				
	**<45**	**45-64**	**65-74**	**≥75**	**Total**
March	158	137	41	23	359
April	330	317	126	190	963
May	202	174	80	133	589
June	679	176	47	58	960
July	3318	1170	352	421	5261
August	1139	535	181	288	2143
September	1300	441	154	165	2060
October	1996	862	277	278	3413
November	7240	3462	1144	1185	13031
December	12764	7083	2529	2751	25127
Total					
All Residents	29126	14357	4931	5492	53906
Independent Residents	28959	13826	4132	2571	49488
Long-Term Care Facility Residents	167	531	799	2921	4418
	**DEATHS**				
	**<45**	**45-64**	**65-74**	**≥75**	**Total**
March	0	1	7	9	17
April	2	16	12	86	116
May	0	4	7	41	52
June	0	1	3	18	22
July	1	8	14	89	112
August	0	8	13	57	78
September	0	2	5	25	32
October	1	5	8	45	59
November	5	16	51	222	294
December	4	45	104	479	632
Total					
All Residents	13	106	224	1071	1414
Independent Residents	11	81	122	307	521
Long-Term Care Facility Residents	2	25	102	764	893

Table S5: Interrupted time series analysis results modelling monthly all-cause death by age group.

Age Group	Coefficient	Coefficient Value	95% Confidence Interval
All	Pre-Pandemic Slope	-1.84	-5.10, 1.43
	Change in Starting Level Post-Pandemic Relative to Pre-	-54.00	-286.98, 178.99
	Post-Pandemic Slope Relative to Pre-	49.96	-4.04, 103.96
45-64	Pre-Pandemic Slope	-0.90	-1.57, -0.24
	Change in Starting Level Post-Pandemic Relative to Pre-	4.37	-21.56, 30.31
	Post-Pandemic Slope Relative to Pre-	5.12	-1.36, 11.59
65-74	Pre-Pandemic Slope	0.45	-0.28, 1.18
	Change in Starting Level Post-Pandemic Relative to Pre-	-20.28	-68.18, 27.62
	Post-Pandemic Slope Relative to Pre-	10.76	1.93, 19.59
≥75	Pre-Pandemic Slope	-0.87	-3.16, 1.42
	Change in Starting Level Post-Pandemic Relative to Pre-	-50.72	-228.17, 126.74
	Post-Pandemic Slope Relative to Pre-	32.62	-7.89, 73.13

All models accounted for autocorrelation with a lag of 1; Pre-Pandemic defined as January 2017 to February 2020; Pandemic defined as March 2020 to December 2020.

Table S6: Interrupted time series analysis modelling monthly cause of death ischemic heart disease by age group.

Age Group	Coefficient	Coefficient Value	95% Confidence Interval
All	Pre-Pandemic Slope	-0.01	-0.62, 0.61
	Change in Starting Level Post-Pandemic Relative to Pre-	-20.72	-46.46, 5.01
	Post-Pandemic Slope Relative to Pre-	5.38	0.66, 10.10
45-64	Pre-Pandemic Slope	<0.01	-0.14, 0.15
	Change in Starting Level Post-Pandemic Relative to Pre-	0.59	-5.45, 6.63
	Post-Pandemic Slope Relative to Pre-	0.26	-0.95, 1.47
65-74	Pre-Pandemic Slope	0.03	-0.18, 0.24
	Change in Starting Level Post-Pandemic Relative to Pre-	-5.19	-11.74, 1.36
	Post-Pandemic Slope Relative to Pre-	2.15	1.28, 3.02
≥75	Pre-Pandemic Slope	-0.02	-0.44, 0.40
	Change in Starting Level Post-Pandemic Relative to Pre-	-16.97	-36.75, 2.81
	Post-Pandemic Slope Relative to Pre-	2.99	-0.25, 6.22

All models accounted for autocorrelation with a lag of 1; Pre-Pandemic defined as January 2017 to February 2020; Pandemic defined as March 2020 to December 2020.
